# A Protocol for Cancer-Related Mutation Detection on Exosomal DNA in Clinical Application

**DOI:** 10.3389/fonc.2020.558106

**Published:** 2020-09-11

**Authors:** Zhe-Ying Wang, Rui-Xian Wang, Xiao-Qing Ding, Xuan Zhang, Xiao-Rong Pan, Jian-Hua Tong

**Affiliations:** Department of Laboratory Medicine and Central Laboratory, Ruijin Hospital, Shanghai Jiao Tong University School of Medicine, Shanghai, China

**Keywords:** exosomes, exosomal DNA, mutation detection, cancer, methodology

## Abstract

**Background:**

Recently, some genomic mutations in exosomal DNA have been found to be related to disease progress and clinical outcomes of patients in several cancers. Unfortunately, the methods for exosome isolation and exosomal DNA analysis are still lack of relevant research to ensure their optimal performance and the comparability. Here we aim to establish a protocol for cancer-related mutation detection on exosomal DNA in clinical application.

**Methods:**

Taking *KRAS* mutation in pancreatic cancer as an example, we tested whether the types of blood samples, the potential factors in the courses of exosome isolation and exosomal DNA preparation, as well as the detail in mutation detection by droplet digital PCR (ddPCR) could influence the exosomal DNA analysis.

**Results:**

We found that the concentration of exosomal DNA from serum was higher than that from plasma, whereas the mutant allele fraction (MAF) of *KRAS* in serum-derived exosomal DNA was obviously lower. The membrane-based method for exosome isolation showed no evident difference in both exosomal DNA yield and *KRAS* MAF from the classical ultracentrifugation method. DNase I pretreatment on exosomes could remove the wild-type DNA outside of exosomes and increase the *KRAS* MAF. PBS might interfere with the effect of DNase I and should not be recommended as resuspension buffer for exosomes if the subsequent experiments would be done with exosomal DNA. Besides, the denaturation of exosomal DNA before droplet generation during ddPCR could effectively improve the total *KRAS* copy number and mutation-positive droplet number.

**Conclusion:**

This study provides some methodological evidences for the selection of the optimal experimental conditions in exosomal DNA analysis. We also suggest a protocol for mutation detection on exosomal DNA, which might be suitable for the clinical testing and could be helpful to the comparison of results from different laboratories.

## Introduction

As the current gold standard for cancer diagnosis, tissue biopsy is usually not convenient, particularly for the patients who cannot undergo surgical resection. Moreover, tumor tissue is very spatial heterogeneous due to uneven distribution of cancer subclones. The genetic alterations may change over time as a result of microenvironmental selection, genomic instability, and response to drug treatments (1). In contrast with tissue biopsy, the detection on circulating DNA is minimally invasive, and may better reflect the overall and real-time tumor burden in cancer patients (2–4). Recently, the presence of double-stranded DNA molecules enclosed in exosomes was described (5). Exosomes are membrane-bound vesicles shed from a large major of cells in the body and are able to mediate intercellular communication by transfer of genetic information (6, 7). It was considered that the membrane of exosomes could protect the nucleic acid material inside from nuclease-induced degradation in the plasma. Thus, the exosomal DNA may exhibit higher molecular weight in comparison with circulating cell-free DNA (cfDNA) (8). So far, exosomal DNA has been approved as high-quality DNA material used for molecular profiling in several cancers. Some tumor-related mutations in exosomal DNA were found to be able to reflect the disease progress and prognosis in patients with pancreatic cancer (PC), colorectal cancer, and so on (9–11).

Despite the great scientific interest on exosomes, the methods of the exosome isolation and subsequent exosomal DNA detection still needs more special studies to ensure their optimal performance. There is no clear consensus on the protocol for mutation detection on exosomal DNA. The procedures in the experiments of exosomal DNA analysis varied across different studies. The experimental results from different laboratories usually could not be directly compared (12). Furthermore, most of the current protocols are not suitable for clinical testing due to the time-consuming work or the requirement of specific infrastructure (13). In order to solve these problems, the methodological research on exosome analysis has got more and more attentions (14, 15). In this study, we tried to establish a protocol for the mutation detection on exosomal DNA and make it available for clinical testing. We took *KRAS* mutation detection in PC patients as an example, and tested whether the potential factors during exosome isolation and exosomal DNA preparation, the selection of blood sample type (plasma or serum), as well as the details in mutation analysis by droplet digital PCR (ddPCR), etc., could influence the mutation detection on exosomal DNA.

## Materials and Methods

### Human Blood Samples

This study was approved by the Ruijin Hospital Ethics Committee and was performed in accordance with the Declaration of Helsinki. All participants provided written informed consent. Whole blood samples from 53 patients with PC were obtained prior to treatment. The clinical information of patients was shown in Table 1. Fifty-three serum samples were isolated from 1.5∼6 ml of peripheral blood collected in Vacutainer Plus plastic serum tubes (BD), while 14 plasma samples were isolated from 1∼4 ml of blood in EDTA tubes (BD). Matched serum and plasma samples were obtained from 14 patients. Seventeen of the unmatched serum samples were mixed and used in the experiments of exosome resuspension buffer comparison and DNase I pretreatment effects. After centrifuged at 1900 × *g* for 10 min at room temperature and then at 16,000 × *g* for 10 min at 4°C, the serum and plasma samples were respectively stored at −80°C until needed.

**TABLE 1:** Patient characteristics.

**Groups**		**Patients for individual sample test**	**Patients for mixed sample test**
Patient number (*n*)		36	17
Age, mean (range) (year)		68 (50∼92)	63 (44∼82)
Gender (percentage)			
	Female	15 (41.7%)	11 (64.7%)
	Male	21 (58.3%)	6 (35.3%)
Clinical Stage (percentage)			
	I	12 (33.3%)	4 (23.5%)
	II	7 (19.4%)	3 (17.6%)
	III	12 (33.3%)	9 (52.9%)
	IV	5 (13.9%)	1 (5.9%)

### DNase Treatment on Plasmid and Agarose Gel Electrophoresis

One plasmid of 4628 bp (constructed in our laboratory) was dissolved in H_2_O, Buffer XE (Qiagen) and PBS (Takara), respectively. In each solvent group, 300 ng of plasmid DNA was treated with 1 unit of DNase I (Thermo Fisher Scientific) for 0, 1, 4 h or overnight. The DNase I digestion was stopped according to manufacturer’s instructions. DNA was separated using 1% agarose gel and stained with GelRed nucleic acid stain (Biotium). Gel images were visualized using Tanon-3500 Gel Image System (Tanon).

### Exosome Isolation and DNase Pretreatment

Two different methods were performed for exosome isolation. For the affinity membrane-based method, exosomes were isolated from serum or plasma samples using ExoEasy Maxi Kit (Qiagen) according to the manufacturer’s protocol, and then treated with 2 units of DNase I at 37°C for the indicated time in 30 μl of reaction system. The DNase I digestion was stopped according to manufacturer’s instructions. For ultracentrifugation, serum or plasma samples were diluted in 13 ml of PBS and centrifuged at 100,000 × *g* for 2 h at 4°C in a SW41 Ti rotor using Optima XPN-100 ultracentrifuge (Beckman Coulter). The exosome pellet was resuspended in Buffer XE (Qiagen) and incubated with 10 units DNase I at 37°C for 4 h in 150 μl of reaction system. After a second ultracentrifugation wash step of 80 min, the resulting exosome pellet was then resuspended in Buffer XE. Exosome enumeration and sizing were carried out using NanoSight NS300 system (Malvern). Images were recorded with detection threshold to 5.

### Exosomal DNA Extraction

DNA was extracted from exosomes with QIAamp DNA Micro Kit (Qiagen) according to the manufacturer’s instructions. DNA quantity was determined using Qubit dsDNA HS Assay Kit and Qubit 3.0 fluorometer (Thermo Fisher Scientific). DNA quality was analyzed by Agilent 2100 system with Agilent DNA 7500 reagent kit (Agilent). The concentration of exosomal DNA was indicated as nanograms per milliliter of serum or plasma samples.

### Detection of KRAS Mutations in Exosomal DNA

The ddPCR platform (Qx200 ddPCR system, Bio-Rad) was used for the detection of *KRAS* mutations in exosomal DNA as the manufacturer’s instruction. The primers (Beijing Genomics Institute) used to amplify a segment in exon 2 of *KRAS* gene (78-bp amplicon) were as follows: Forward primer: 5′-GCCTGCTGAAAATGACTGAAT-3′; Reverse primer 5′-GCTGTATCGTCAAGGCACTCT-3′. Multiple hotspot mutations within codon 12 and codon 13 of *KRAS* gene exon 2 were detected with a pair of drop-off and reference probes as described (16). The sequences of these two probes (Thermo Fisher Scientific) were 5′-(6-FAM)-CTACGCCACCAGCT-(MGB NFQ)-3′ and 5′-(VIC)-CAACTACCACAAGTTT-(MGB NFQ)-3′, respectively. Twenty microliter reaction solutions were prepared with dUTP-free Supermix for probes (Bio-Rad), 900 nM of primers, 250 nM of hydrolysis probes and at least 0.5 ng of exosomal DNA. The amplification was performed under the following programs: 95°C for 10 min, 40 cycles of (94°C for 30 s, 60°C for 60 s), 98°C for 10 min. The results were analyzed by the Quanta-Soft Analysis Pro software (Bio-Rad). For denaturation-enhanced ddPCR (17), the reaction solutions were placed in a T100 Thermal Cycler (Bio-Rad) for DNA denaturation at 95°C for 1 min before droplet generation, in order to double the number of positive droplets obtained from a given amount of input DNA.

### Statistical Analyses

Statistical analyses were performed using the SPSS Version 23 software (IBM). Continuous data were compared using Wilcoxon test. A *p*-value < 0.05 was considered as significant.

## Results

### PBS Is Not Recommended as an Exosome Resuspension Buffer for Subsequent DNA Extraction

**FIGURE 1:**
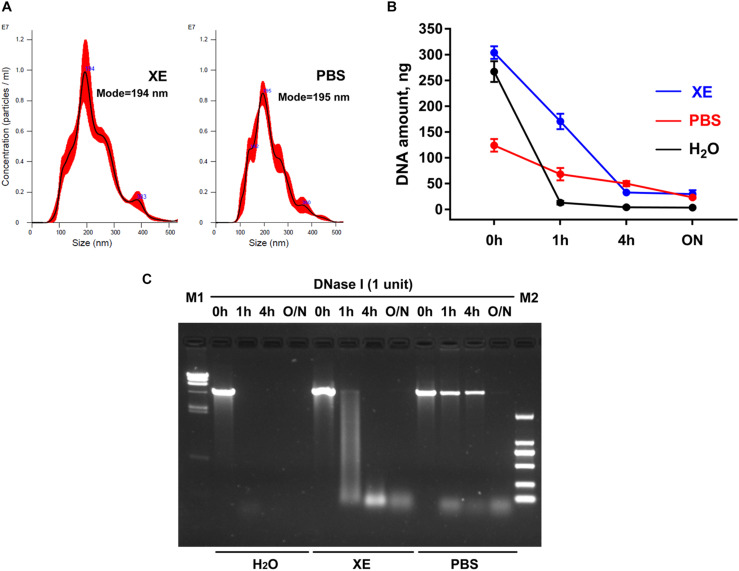
Effects of exosome resuspension buffers on subsequent DNA extraction. **(A)** NanoSight analysis for particle number and size distribution of exosomes suspended in PBS and Buffer XE. **(B)** Change of DNA content in three different solvents after DNase I digestion for the indicated time. The results were repeated for at least five times. **(C)** Agarose gel electrophoresis of plasmid DNA in three different solvents. M1, λ-*Hin*dIII digest DNA marker; M2, DL2000 DNA marker; O/N, overnight.

At present, PBS is a commonly used resuspension buffer for exosomes obtained after ultracentrifugation. However, when we use the membrane-based method for exosome isolation, the manufacturer normally provides Buffer XE as the resuspension buffer for exosomes. Are there any differences between these two buffers in the studies on exosomes and exosomal DNA? Here, by the nanoparticle tracking analysis, we first demonstrated that under the same condition, the particle number and the size distribution of the exosomes suspended in the above two buffers were similar (Figure 1A). Considering the reports that the exosomes obtained through the existing isolation methods might contain some impurities, such as the potential contamination of external DNA associated with the outer membrane of exosomes (18), we then tried to remove the external DNA with DNase I treatment before proceeding with the extraction of exosomal DNA. In order to compare the effects of DNase I on the external DNA in different resuspension buffers, we used linearized plasmid DNA to mimic the situation of DNA not incorporated into the exosome membrane. We compared the quantification results of plasmid DNA dissolved in three different solutions (PBS, H_2_O and Buffer XE). The same amount of plasmid DNA was put in equal volume of these solutions, respectively. The results showed that the concentration of the plasmid dissolved in PBS was lower than that in H_2_O (*p* = 0.008, Wilcoxon test) or Buffer XE (*p* = 0.008, Wilcoxon test), suggesting that PBS could affect the solubility of DNA (Figure 1B). Next, we further compared the efficiency of DNase I on plasmid DNA dissolved in different buffers. The concentration change of the plasmid along with DNase I digestion was shown in Figure 1B. The agarose gel electrophoresis illustrated that the plasmid DNA was completely degraded into small fragments in H_2_O for over 1 h or in Buffer XE for over 4 h. In contrast, the plasmid DNA in PBS could not be thoroughly degraded by DNase I until overnight (Figure 1C). We observed that in PBS solution, one high molecular weight band was always present in the original position (around 4.4 kb) after 4 h of DNase I digestion. Such incomplete digestion could be related with the reduction of DNA solubility in PBS. The similar phenomenon was also very likely to exist during the exosomal DNA extraction. For the exosomes suspended in PBS, the DNA associated to the outer membrane of exosomes may not be effectively removed by DNase in a limited period of time. Therefore, it seemed that PBS was not a suitable buffer for exosomal DNA extraction. Besides, though DNase I activity was better in H_2_O, the H_2_O was usually not selected for exosome resuspension because its non-physiological salt concentration might affect the integrity and biological activity of exosomes. To ensure the purity of the exosomal DNA extracted, we chose the Buffer XE to suspend the exosomes in our subsequent experiments.

### DNase I Pretreatment on Exosomes Benefits the Mutation Detection on Exosomal DNA

**FIGURE 2:**
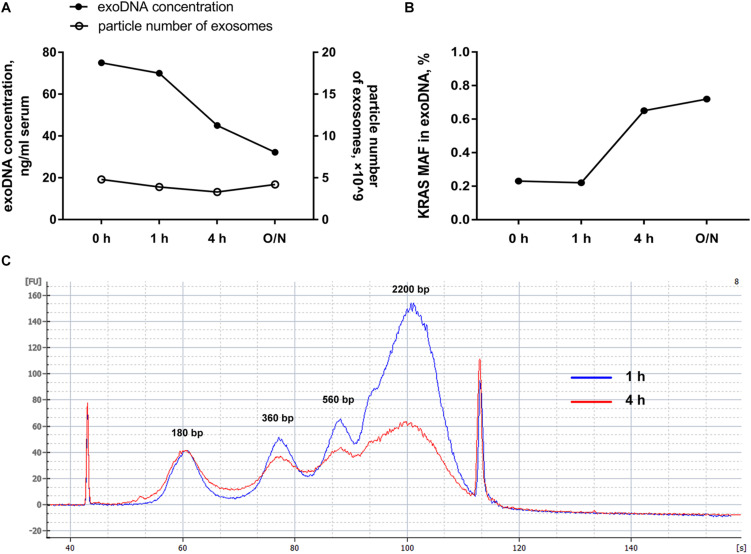
Effects of DNase I pretreatment on exosomes. **(A)** Particle number of exosomes with or without DNase I pretreatment for the indicated time and the amount of exosomal DNA from above exosomes. **(B)** MAF of *KRAS* in DNA extracted from exosomes before and after DNase I pretreatment for the indicated time. **(C)** Agilent 2100 system analysis of size distribution of DNA extracted from exosomes after DNase I digestion for 1 and 4 h. O/N, overnight.

In order to further explore whether the external DNA associated with the outer membrane of exosomes could influence the mutation detection, hotspot mutations of *KRAS* in codon 12 and codon 13 within PC patients were detected by ddPCR. Along with DNase I pretreatment, we found that the total amount of exosomal DNA was obviously reduced and the particle number of the isolated exosomes was almost unchanged (Figure 2A). But the mutant allele fraction (MAF) of *KRAS* was increased after DNase I treatment for over 4 h (Figure 2B). The Agilent 2100 system analysis demonstrated that after the elimination of external DNA by DNase I for 4 h, the exosomes showed a strong reduction in DNA fragments larger than 1.5 kb (Figure 2C). The main peaks of exosomal DNA that we extracted were around 180, 360, 560, and 2200 bp range in size. These results suggested that the majority of DNA associated with the outer membrane of exosomes was the wild-type DNA with a larger size, which was most probably from non-tumor cells. The DNase I pretreatment could effectively eliminate the interference of the external DNA outside of exosomes, and thus improve the mutation detection.

### Effects of Different Exosome Isolation Methods on Subsequent DNA Analysis

**FIGURE 3:**
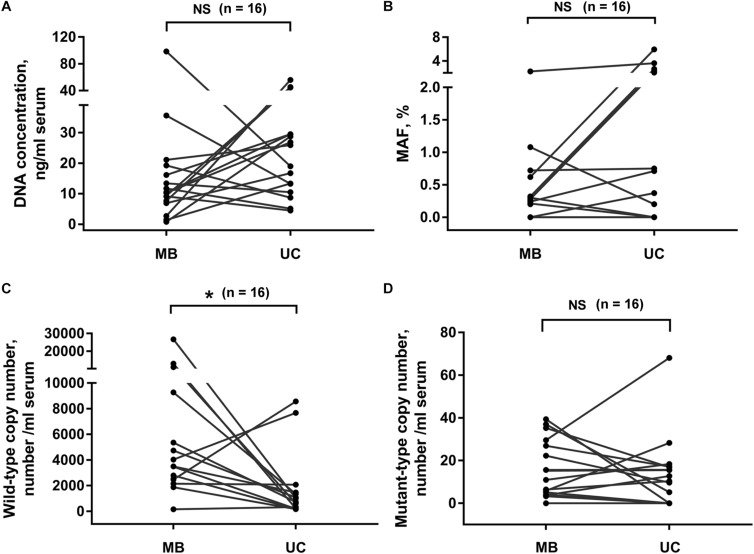
Effects of different exosome isolation methods on subsequent DNA analysis. Comparison of DNA concentration **(A)**, *KRAS* MAF **(B)** and the copy number of wild-type **(C)**, and mutant-type **(D)** *KRAS* in DNA extracted from exosomes isolated by ultracentrifugation and membrane-based method. Serum samples were collected from 16 PC patients. UC, ultracentrifugation; MB, membrane-based method. *p < 0.05; NS, not significant.

Next, we compared the DNA extracted from exosomes isolated by membrane-based method and ultracentrifugation. Sixteen serum samples were collected from PC patients. The same volume of serum sample from the same patient was used for exosome isolation by these two methods, respectively. Our results showed that the exosomal DNA concentration in membrane-based method group was similar with that in ultracentrifugation group (Figure 3A, *p* = 0.159, Wilcoxon test). The detection rate of *KRAS* mutation was 68.8% (11/16) in exosomes isolated by ultracentrifugation and 75% (12/16) in exosomes isolated by membrane-based method, respectively. The concordance rate of *KRAS* mutation status (mutant or wild-type) was 81.3% (13/16) (Supplementary Table 1). There was no significant difference in either *KRAS* MAF (Figure 3B, *p* = 0.054, Wilcoxon test) or mutant *KRAS* copy number (Figure 3C, *p* = 0.525, Wilcoxon test) between the two groups. In addition, the wild-type *KRAS* copy number in the membrane-based method group was slightly higher than that in ultracentrifugation group (Figure 3D, *p* = 0.035, Wilcoxon test).

### Comparison of Exosomal DNA Extracted From Serum and Plasma Samples

**FIGURE 4:**
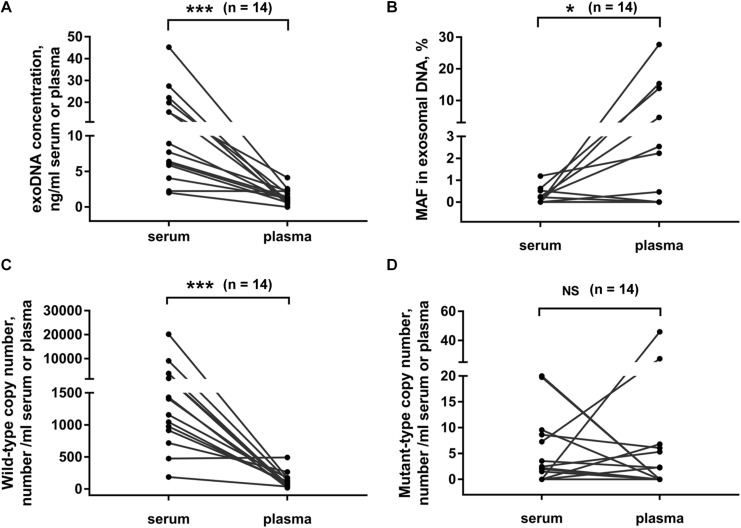
Comparison of exosomal DNA extracted from serum and plasma. DNA concentration **(A)**, MAF of *KRAS* **(B)** and copy number of wild-type **(C)**, and mutant-type **(D)** *KRAS* in exosomal DNA from serum or plasma of PC patients. Matched serum and plasma samples were collected from each patient at the same time point in 14 PC patients. *p < 0.05; ***p < 0.001; NS, not significant.

Usually, both serum and plasma samples could be used for exosome isolation. Here we compared the exosomal DNA extracted from serum- and plasma-derived exosomes. In 14 PC patients, matched serum and plasma samples were collected from each patient at the same time point. We found that the concentration of exosomal DNA extracted from serum was higher than that from plasma (Figure 4A, *p* < 0.001, Wilcoxon test). The detection rate of *KRAS* mutation in exosomal DNA was 42.9% (6/14) in serum and 50% (7/14) in plasma samples. The concordance rate of *KRAS* mutation status in serum- and plasma-derived exosomal DNA was 64.3% (9/14) (Supplementary Table 2). However, *KRAS* MAF in exosomal DNA derived from serum was obviously lower than that from plasma (Figure 4B, *p* = 0.027, Wilcoxon test). Wild-type *KRAS* copy number in serum exosomes was higher than that in plasma exosomes (Figure 4C, *p* < 0.001, Wilcoxon test), but no significant difference was observed in mutant *KRAS* copy number (Figure 4D, *p* = 0.972, Wilcoxon test).

### Denaturation of Exosomal DNA Increases the Number of Mutation-Positive Droplets During ddPCR Analysis

**FIGURE 5:**
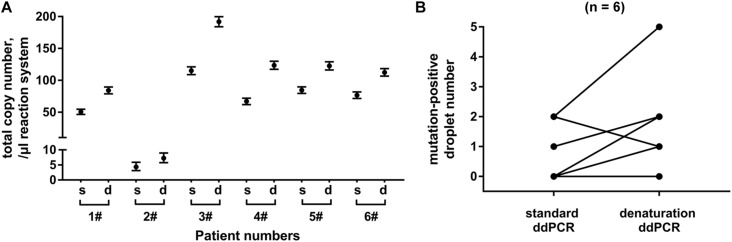
Analysis of denaturation-enhanced ddPCR on exosomal DNA. Total copy number of *KRAS* **(A)** and number of mutation-positive droplets **(B)** detected by standard ddPCR and denaturation-enhanced ddPCR, respectively. Exosomal DNA samples were obtained from serum of 6 PC patients. “s” indicates standard ddPCR and “d” indicates denaturation-enhanced ddPCR.

Finally, we compared denaturation-enhanced ddPCR with standard ddPCR in *KRAS* mutation detection. Exosomal DNA samples were obtained from serum of six PC patients. We showed that with DNA denaturation before droplet formation, the total number of *KRAS* copies detected in all six patients was effectively improved. Denaturation-enhanced ddPCR detection on exosomal DNA resulted in an about 1.65 (range: 1.44∼1.84) fold increase in total *KRAS* copy number compared with standard ddPCR (Figure 5A). In four out of six DNA samples tested, there was an increased number of mutation-positive droplets when denaturation-enhanced ddPCR was employed (Figure 5B).

## Discussion

**FIGURE 6:**
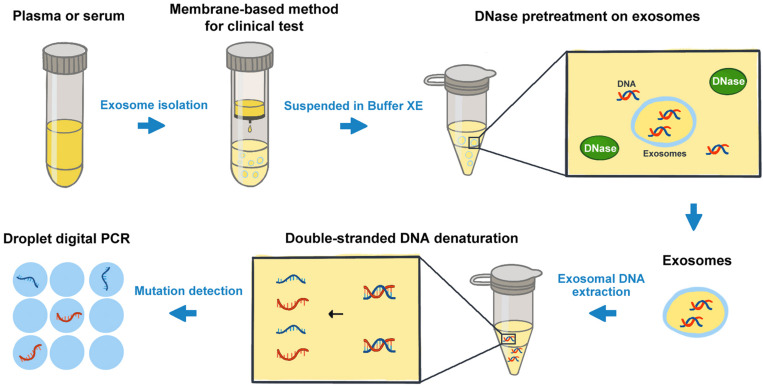
A brief work-flow chart for the mutation detection on exosomal DNA from cancer patients.

The analysis of cancer cell-derived exosomes has been one of the most exciting and rapidly advancing fields in oncology research (19, 20). Recently, the presence of double-stranded DNA molecules enclosed in exosomes was described. The mutant exosomal DNA from blood samples was found to be related to the disease progress and clinical outcomes of patients in several cancers (10, 21). Bernard et al. (22) demonstrated that the monitoring of *KRAS* mutation in exosomal DNA could provide both predictive and prognostic information relevant toward therapeutic stratification in PC. Wang et al. (23) found that the somatic mutation screens of exosomal DNA might be used for the diagnosis and preoperative assessment of pheochromocytomas and paragangliomas. Though the great progress has been made in the related research in recent years, the understanding of the composition and properties of extracellular vesicles (EVs) has been limited because of the heterogeneity of EVs (24). It is now increasingly clear that “exosomal” samples contain a heterogeneous mixture of small EVs. The methodology studies on exosomal DNA analysis still need some particular concerns (25), especially on the processes of exosome isolation and the subsequent DNA detection, etc. In this study, we took *KRAS* mutation detection in PC patients as an example, and compared the effects of some potential factors on the exosomal DNA analysis. The advantages and limitations of certain procedures were highlighted in Table 2. And we suggested a protocol for cancer-related mutation detection of exosomal DNA in clinical application (Figure 6).

**TABLE 2:** Characteristics of certain procedures in mutation detection on exosomal DNA.

**Procedures**	**Characteristics**	**Recommendation**
Types of blood samples		
Plasma	Low DNA yield, exact MAF	★★★
Serum	High DNA yield, relatively low MAF	★★
Exosome isolation methods		
Ultracentrifugation	Current golden standard, requirement for special equipment, time-consuming process	★★
Membrane-based method	Rapidness, convenience, suitableness of clinical detection, need for more validation	★★★
Exosome suspension buffers		
PBS	Reduction in DNA solubility and DNase I efficiency	✩
Buffer XE	None of the above	★★★
DNase I pretreatment		
Treated	Enrichment of exosomal DNA	★★★
Untreated	Interference from wild-type DNA outside of exosomes	✩
ddPCR testing methods		
Standard ddPCR	Absolute quantification, high sensitivity	★★
Denaturation-enhanced ddPCR	Increase in droplets containing nucleic acid, higher sensitivity and precision	★★★

*ddPCR, digital droplet PCR; MAF, mutant allele fraction. ★★★, highly recommended; ★★, recommended; ✩, not recommended.*

Firstly, prior to DNA acquisition, exosomes could be isolated from different types of blood samples such as plasma and serum. Our results indicated that the concentration of exosomal DNA extracted from serum was much higher than that from plasma. However, the MAF of *KRAS* in serum exosomal DNA was obviously lower. In comparison with plasma samples, a large number of platelets are activated in serum during the coagulation process. According to the literature, platelet activation will result in release of EVs, including microvesicles of 100 nm to 1 mm and exosomes measuring 40–100 nm in diameter (26). Exosomal DNA extracted from serum samples might include wild-type DNA of the exosomes produced during clotting by platelets (27). Thus, the mutation detection on serum-derived exosomal DNA was more likely to be affected and show obviously lower *KRAS* MAF. However, no significant difference was observed in mutant *KRAS* copy number. So we considered that both serum- and plasma-derived exosomal DNA could be used for *KRAS* mutation detection in PC. But it should be noted that the detection on exosomal DNA from plasma may need sufficient blood to ensure the adequate template for subsequent analysis. For serum-derived exosomes, though the amount of DNA obtained was relatively high, false-negative results may happen in the cases with very low MAF.

Secondly, exosomes could be isolated by different methods including ultracentrifugation, co-precipitation, membrane affinity methods, etc. (28). So far, ultracentrifugation is considered as one of the most classic and reliable methods for exosome isolation and is widely used by many researchers. But it is difficult to be implemented in clinical routine due to the inconvenient procedures and the requirement of specific infrastructure (13). In comparison with the ultracentrifugation method, the membrane-based method takes the advantages of rapidness, convenience and independence from special equipment, and is more suitable for clinical testing. In the present study, we found that for *KRAS* mutation detection on exosomal DNA, the membrane-based method showed no obvious difference from the classical ultracentrifugation method. In order to benefit the possible clinical application of exosomal DNA detection, here we recommended using the membrane-based method in priority.

In addition, the resuspension buffer for exosomes and the external DNA associated with the outer membrane of exosomes may also influence the mutation detections on exosomal DNA obtained (29). Here we compared the two different buffers used for exosome resuspension. Our results indicated that PBS could reduce the solubility of DNA and the efficiency of DNase I digestion, whereas Buffer XE did not. Thus, we were not inclined to recommend PBS as a resuspension buffer for exosomes if the subsequent experiments would be conducted with the exosomal DNA. Besides, we found that the DNA extracted from exosomes pretreated with DNase I showed a strong reduction in DNA fragments greater than 1.5 kb in size and an enrichment of DNA between 100 bp and 1.5 kb. The main peaks of exosomal DNA were around 180, 360, 560, and 2200 bp in size, and were as similar as reported in relevant studies (5, 30). Of note, several researchers also revealed the presence of high molecular weight (>10 kb) DNA in exosomes (21, 31). The assessment on the size distribution of exosomal DNA may require further research.

Finally, we also improved the mutation detection method on the exosomal DNA samples according to the denaturation-enhanced ddPCR described by Fitarelli-Kiehl et al. (17). The authors proved that for genomic DNA and cfDNA, 95°C for 1 min could enable the denatured single DNA strands to partition into droplets and theoretically double the number of positive events during ddPCR, leading to higher sensitivity and precision of this detection method. In our study, we found similar results on exosomal DNA by denaturation-enhanced ddPCR in comparison with standard ddPCR. The number of total *KRAS* copies and mutation-positive droplets were both increased when denaturation-enhanced ddPCR was employed. Therefore, we considered that DNA denaturation before droplet formation could obviously benefit the mutation detection on exosomal DNA, especially for the samples with very low DNA content or very low MAF.

## Conclusion

In conclusion, our study provides some methodological evidences for the selection of the optimal experimental conditions in exosomal DNA analysis. We recommend a protocol for mutation detection on exosomal DNA, which might be suitable for the clinical testing and could be helpful to the comparison of results from different laboratories. This study could benefit the future laboratory research on exosomal DNA in cancer patients and help to facilitate its translation to clinical practice.

## Data Availability Statement

The raw data supporting the conclusions of this article will be made available by the authors, without undue reservation.

## Ethics Statement

The studies involving human participants were reviewed and approved by the Ruijin Hospital Ethics Committee. The patients/participants provided their written informed consent to participate in this study.

## Author Contributions

Z-YW, X-RP, and J-HT designed the study, supervised the research, interpreted the data, and revised the manuscript. Z-YW, R-XW, X-QD, and XZ conducted the experiments, analyzed the data, and wrote the manuscript. All authors contributed to the article and approved the submitted version.

## Conflict of Interest

The authors declare that the research was conducted in the absence of any commercial or financial relationships that could be construed as a potential conflict of interest.
