# Supplementary material for: A Protocol for Cancer-Related Mutation Detection on Exosomal DNA in Clinical Application
Source: Front Oncol. 2020 Sep 11;10:558106. doi: 10.3389/fonc.2020.558106 (PMC7518026; doi:10.3389/fonc.2020.558106)
Supplement: Supplementary file 1 [file Data_Sheet_1.docx]

Supplementary Material

# Supplementary Tables

**Supplementary Table 1** *KRAS* mutation status in DNA extracted from exosomes isolated by ultracentrifugation and membrane-based method

| **Groups** | | **Membrane-based method** | | |
| --- | --- | --- | --- | --- |
|  |  | ***KRAS* mutant** | ***KRAS* wild-type** | **Total** |
| **Ultra-**  **centrifugation** | ***KRAS* mutant** | 10 | 1 | 11 |
|  | ***KRAS* wild-type** | 2 | 3 | 5 |
|  | **Total** | 12 | 4 | 16 |

**Supplementary Table 2** *KRAS* mutation status in exosomal DNA extracted from serum and plasma of pancreatic cancer patients

| **Groups** | | **Exosomal DNA in serum** | | |
| --- | --- | --- | --- | --- |
|  |  | ***KRAS* mutant** | ***KRAS* wild-type** | **Total** |
| **Exosomal DNA**  **in plasma** | ***KRAS* mutant** | 4 | 3 | 7 |
|  | ***KRAS* wild-type** | 2 | 5 | 7 |
|  | **Total** | 6 | 8 | 14 |
